# pH responsive superporogen combined with PDT based on poly Ce6 ionic liquid grafted on SiO_2_ for combating MRSA biofilm infection

**DOI:** 10.7150/thno.42922

**Published:** 2020-03-26

**Authors:** Chaoli Wang, Peng Chen, Youbei Qiao, Yuan Kang, Chaoren Yan, Zhe Yu, Jian Wang, Xin He, Hong Wu

**Affiliations:** 1 Department of pharmacy, Air Force Medical University, Xi'an, 710032, Shaanxi Province, China; 2 Department of physics, Northwest University of Technology, Xi'an, 710032, Shaanxi Province, China; 3 Department of Thoracic Surgery, Tangdu Hospital, Air Force Medical University, Xi'an, 710032, Shaanxi Province, China

**Keywords:** pH-responsive, polyionic liquids, superporogen, photosensitizer, SiO_2_, MRSA biofilm

## Abstract

**Background**: Biofilm infection caused by multidrug-resistant bacteria is difficult to eradicate by conventional therapies. Photodynamic therapy (PDT) is an effective antibacterial method for fighting against biofilm infection. However, the blocked photosensitizers outside of biofilm greatly limit the efficacy of PDT.

**Methods**: Herein, a novel acid-responsive superporogen and photosensitizer (SiO_2_-P_Ce6-IL_) was developed. Because of the protonation of the photosensitizer and the high binding energy of the polyionic liquid, SiO_2_-P_Ce6-IL_ changed to positive SiO_2_-P_IL_^+^ in an acidic microenvironment of biofilm infection. SiO_2_-P_IL_^+^ could combine with negatively charged extracellular polymeric substances (EPS) and create holes to remove the biofilm barrier. To strengthen the interaction between SiO_2_-P_IL_^+^ and EPS, SiO_2_-P_IL_^+^ of high charge density was prepared by grafting the high-density initiation site of ATRP onto the surface of the SiO_2_ base.

**Results**: Due to the rapid protonation rate of COO^-^ and the strong binding energy of SiO_2_-P_IL_^+^ with EPS, SiO_2_-P_Ce6-IL_ could release 90% of Ce6 in 10 s. With the stronger electrostatic and hydrophobic interaction of SiO_2_-P_IL_^+^ with EPS, the surface potential, hydrophobicity, adhesion and mechanical strength of biofilm were changed, and holes in the biofilm were created in 10 min. Combining with the release of photosensitizers and the porous structure of the biofilm, Ce6 was efficiently concentrated in the biofilm. The *in vitro* and *in vivo* antibacterial experiments proved that SiO_2_-P_Ce6-IL_ dramatically improved the PDT efficacy against MRSA biofilm infection.

**Conclusion**: These findings suggest that SiO_2_-P_Ce6-IL_ could rapidly increase the concentration of photosensitizer in biofilm and it is an effective therapy for combating biofilm infection.

## Introduction

It is well known that bacteria shielded by biofilm are difficult to eradicate 1-3. Once the biofilm forms, the resistance of the bacteria is increased by 1000~1500 times versus that of individual bacteria 4, 5. Especially for the emergence and rapid spread of multidrug-resistant pathogens, biofilm infection is difficult to treat with conventional antibiotics 6, 7. Hence, it is urgent to develop new therapeutic agents or strategies against drug-resistant bacterial biofilm infection.

Photodynamic therapy (PDT) based on photosensitizers to generate ROS through type I and type II oxidative reaction damages the structure and function of the biomolecule 8. Compared with antibiotics, photosensitizers work through a multi-targeted oxidation mechanism against it which is impossible to develop resistance, and they also have excellent inhibitory activity against drug-resistant bacteria 9-11. However due to the electrostatic repulsion between photosensitizers and EPS (the main negative components in biofilm) 12, most photosensitizers are difficultly concentrated in biofilm. Because ^1^O_2_ exhibits a short diffusion distance (approximately 10 nm) and short lifetime (3.5 μs) 13,14, the photosensitizers irradiated outside of the biofilm access the thick layer of biofilm (approximately 40 µM) with difficulty 15 and cannot kill the bacteria in the biofilm. In recent years, multifunctional photodynamic antibacterial systems have been used to reduce the repulsive interaction between the photosensitizers and EPS. For example, cationic polymers have been used to neutralize the negatively charged EPS and weaken the repulsion 16, 17. However, the photosensitizers connected with cationic polymers via covalent bonding are immobilized on the EPS, and the lethal photosensitization occurs mainly in the outermost layers of biofilm 18. The efficacy of PDT against biofilm has not been improved.

Due to the encapsulation of EPS, the infection site of biofilm is hypoxia, and the anaerobic glycolysis increase markedly. This result in the acidic microenvironment of the biofilm infection 19. So, the acid-sensitive covalent bond is used to solve the problem of photosensitizer release 20, 21. However, our previous research found that the traditional acid sensitive bond such as hydrazone takes approximately 24-48 h to break above 80%. Photosensitizers are difficult to release. Unreleased photosensitizers cannot penetrate through biofilm and concentrate in biofilm. Because the bacteria grow rapidly and can reproduce in 10-20 min 22, the low enrichment efficiency of photosensitizers in biofilm easily miss the best treatment time. Therefore, a new strategy should be explored to overcome the release rate of photosensitizers.

Ionic liquids comprise cations and non- covalently connected anions 23, 24. Compared with the strong covalent bonds, the unique interaction of ionic liquids is relatively weak and more likely to exhibit rapid dissociation behavior 25, 26. Thus the carboxyl groups of photosensitizers as the anion of ionic liquids could solve the release problem of photosensitizers caused by the linkage of covalent bonds. More importantly, as a “designed” substance, the structure of cations can be designed to have a specific function 27. For instance, changing the carbon chain length at the N3 position of imidazolium cation is expected to destroy the biofilm integrity 28, 29. Therefore, the combination of ionic liquids and photosensitizers can not only solve the release problem but also eliminate the biofilm barrier and then rapidly increase the concentration of photosensitizers in the biofilm.

**Figure 1:**
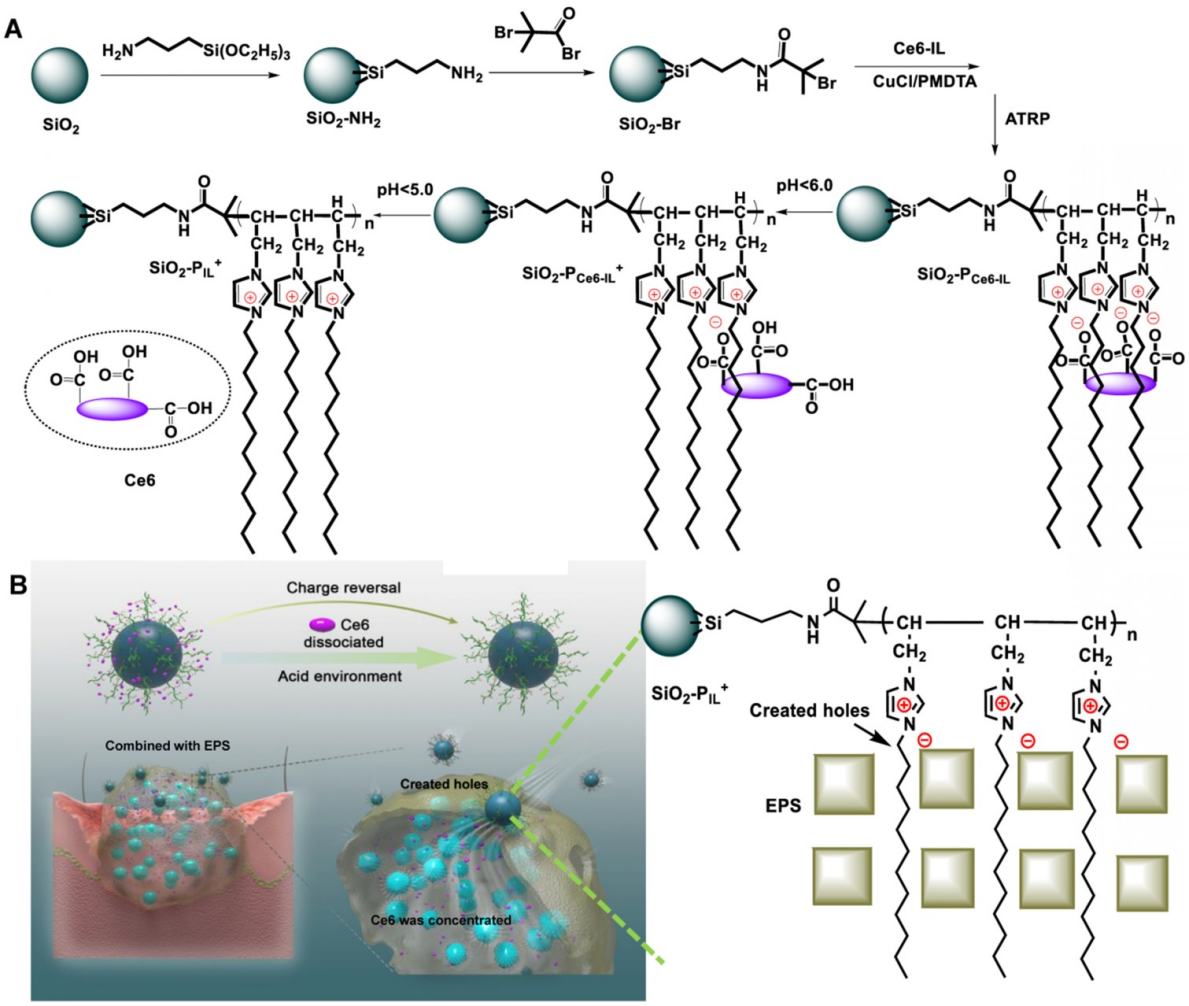
** (A)** Schematic illustrating the synthetic route and responsive properties of SiO_2_-P_Ce6-IL_. **(B)** The SiO_2_-P_IL_^+^ bonded with negatively charged EPS and created holes in the biofilm and then the Ce6 was concentrated.

In this work, Ce6 with three COO^-^ as the anions and 1-vinyl imidazole with dodecyl as the cation and the pH-responsive Ce6 ionic liquid (Ce6-IL) were assembled by an anion exchange reaction. SiO_2_ nanoparticles were introduced to graft different concentration initiation sites of atom transfer radical polymerization (ATRP) and control the density of Ce6-IL polymers. In the physiological environment, the hole-forming ability of SiO_2_-P_Ce6-IL_ was shielded to reduce the damage to normal tissue. In the acidic environment of biofilm infection, Ce6 was protonated and released. Meanwhile, the SiO_2_-P_Ce6-IL_ reversed to SiO_2_-P_IL_^+^ for bonding with negatively charged EPS and was hammered into biofilm to create holes (Figure 1). For maximum punch capacity, SiO_2_-P_Ce6-IL_ of high charge density was prepared. After the Ce6 concentrated in biofilm, the illumination of 660 nm was used to produce ROS to kill MRSA.

## Results and Discussion

### The selection of anion and cation

The photosensitizer Ce6, which has three COO^-^ and can be protonated in an acidic environment, was selected as the anion. However, because everything inside the biofilm occurs in a gradient (nutrients, oxygen, and pH itself), the biofilm did not have a unique pH and not all of three COO^-^ could be protonated in weak acid environment. Other strategies should be used to ensure that photosensitizers can be released under both weakly and strongly acidic conditions. In this work, the cation was designed to enhance the interaction between cations and EPS. When the designed cation was combined with EPS, the stronger interaction made it dissociate with Ce6 and then accelerated the release of Ce6. The structure of the cation was designed as follows: the 1-viny-3-dodecyl-imidazole (IL) that could bond with EPS and destroy biofilm integrity 30, 31 was selected as the cation. To estimate the interaction energy of 1-vinyl-3-dodecyl imidazole and EPS, the binding energy (Δ*Ε*) of 1-vinyl-3-dodecyl imidazole and polysaccharide poly-β-1, 6-N-acetylglucosamine (PNAG) 32, which is the main factor of EPS that maintains the integrity of the biofilm structure, was measured by molecular dynamic simulation (Figure S1). As shown in Table 1, the Δ*Ε* of 1-vinyl-3-dodecyl imidazole with PNAG (Δ*Ε*_1_) was -6.839135 Kcal/mol, and when the 1-vinyl-3-dodecyl imidazole was polymerized to form polycation (P_IL_^+^), the Δ*Ε*_2_ of P_IL_^+^ with PNAG was -23.442899 Kcal/mol and increased significantly. The stronger interaction of P_IL_^+^ and PNAG will accelerate Ce6 release even in a weakly acidic environment (pH less than pKa of Ce6) and can provide a great destructive power in destroying the structure of biofilm.

### Synthesis and characterization of Ce6-IL

As reported, the pH of the MRSA biofilm was approximately 5.5, 5.0 and even lower 33. Thus, Chlorin e6 (Ce6) with three COOH was used as the model. Under the alkaline conditions, the three COOH of Ce6 changed to COO^-^ and were assembled into Ce6-IL with 1-vinyl-3-dodecyl-imidazole (Figure S2). Because of the different modifications, the ionization constants (pKa) of Ce6 were varied. For example, the pKa of Ce6 alone was 4.4, 4.7, and 4.8, respectively. After conjugation with aptamers, the pKa of three Ce6 would occur between 6.5 and 8.5 34. In this work, when Ce6 was assembled with imidazole ionic liquid (Ce6-IL), the pKa was 4.6, 5.7 and 6.7, respectively (Figure S3). The other characterization of Ce6-IL has been shown in our previous research 35.

### Synthesis of SiO_2_-P_Ce6-IL_

**Figure 2:**
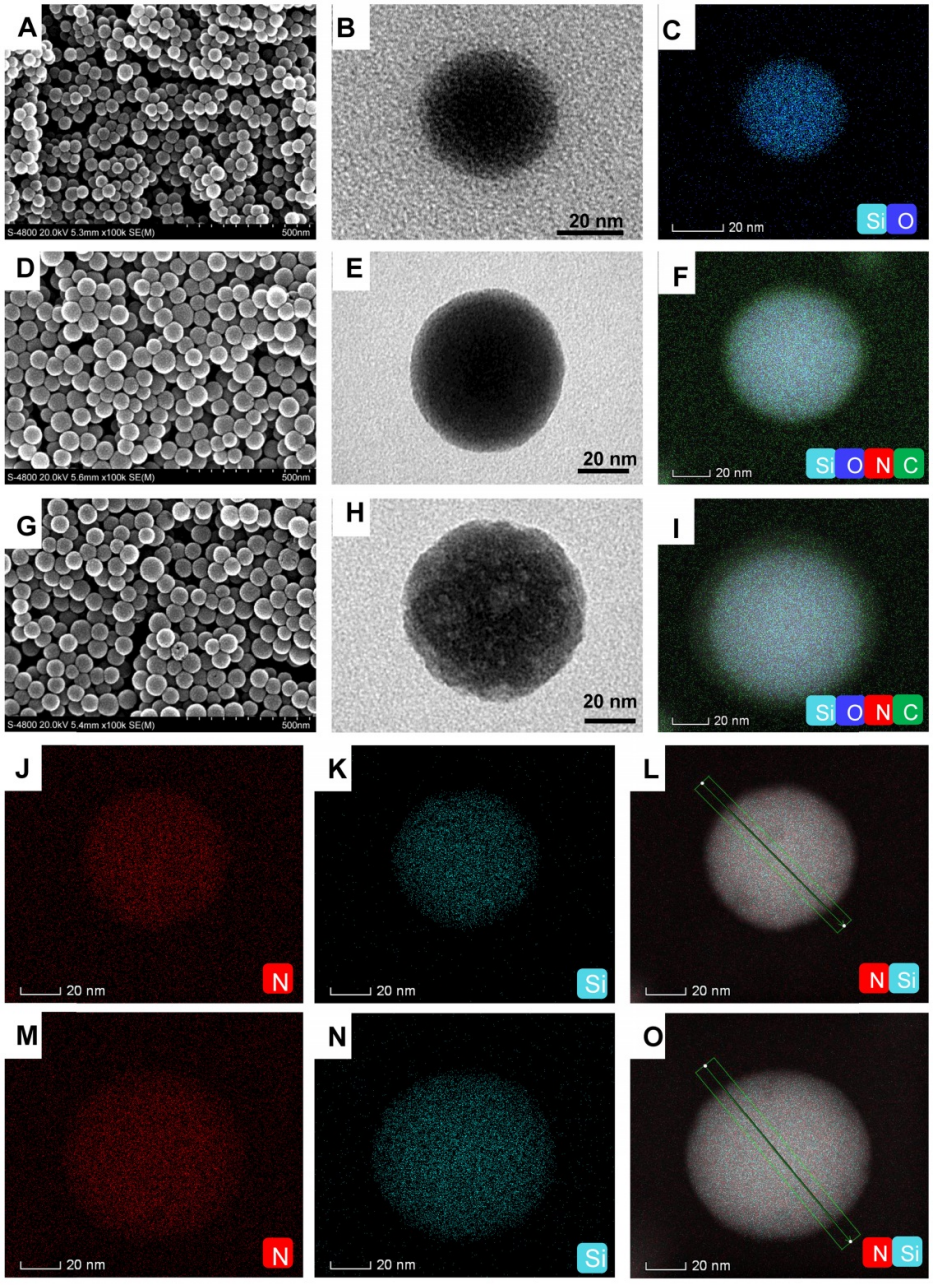
** The morphology and element analysis. (A)** SEM of SiO_2_.** (B)** TEM of SiO_2_. **(C)** The Si and O analysis of SiO_2_. **(D)** SEM of SiO_2_-P_Ce6-IL1_. **(E)** TEM of SiO_2_-P_Ce6-IL1_. **(F)** The Si, O, C and N analysis of SiO_2_-P_Ce6-IL1_. **(G)** SEM of SiO_2_-P_Ce6-IL2_. **(H)** TEM of SiO_2_-P_Ce6-IL2_. **(I)** The Si, O, C and N analysis of SiO_2_-P_Ce6-IL2_. **(J)** The location analysis of N in SiO_2_-P_Ce6-IL1_. **(K)** The location analysis of Si in SiO_2_-P_Ce6-IL1_. **(L)** The location of Si and N in SiO_2_-P_Ce6-IL1_. **(M)** The location analysis of N in SiO_2_-P_Ce6-IL2_. **(N)** The location analysis of Si in SiO_2_-P_Ce6-IL2_. **(O)** The location of Si and N in SiO_2_-P_Ce6-IL2_.

The computer simulation result showed that the polymerized Ce6-IL could strengthen the interaction of 1-vinyl-3-dodecyl imidazole and PNAG. Furthermore, because the chain length and positive charge density of polycations had an important influence on antimicrobial properties 36, 37, P_Ce6-IL_ of the different chain lengths and charge densities was grafted onto the SiO_2_ by regulating the concentration of the reactive sites (Br) of ATRP. The grafting content of Br on the SiO_2_ was 1.07% and 7.52% (Figure S4). After adding Ce6-IL and initiator CuCl, the site-specific *in situ* polymerization was induced. Because the same molar of monomer (Ce6-IL) was used, fewer initiation sites of Br on the SiO_2_ (1.07%) will lead to low charged density P_Ce6-IL1_ with long polymer chains on the SiO_2_ (SiO_2_-P_Ce6-IL1_). In contrast, high charged density P_Ce6-IL2_ with short polymer chains on the SiO_2_ (SiO_2_-P_Ce6-IL2_) were prepared by highly concentrated Br (7.52%). The SEM and TEM results showed that the size of SiO_2_ was approximately 40 nm (Figure 2 A, B, C). After polymerization, the size of SiO_2_-P_Ce6-IL1_ and SiO_2_-P_Ce6-IL2_ was approximately 60 (Figure 2 D, E, F) and 70 nm (Figure 2 G, H, I). The dynamic light scattering (DLS) results indicated that SiO_2_-P_Ce6-IL1_ and SiO_2_-P_Ce6-IL2_ have excellent stability in PBS (Figure S5). Although fewer reactive sites would result in a longer polymer chain and larger particle size, the size of SiO_2_-P_Ce6-IL1_ was smaller than SiO_2_-P_Ce6-IL2_. This may be caused by the longer polycationic polymer chains partly entwined with SiO_2_ nanoparticles.

The element analysis showed that the N of P_Ce6-IL_ appeared on the SiO_2_ after polymerization. The location of the Si and N was further analyzed by spherical aberration corrected transmission electron microscope (ACTEM, Figure 2J, L, N, O). The result showed that the N was on the surface of SiO_2_ (Figure 2M, P and Figure S6).

**Figure 3:**
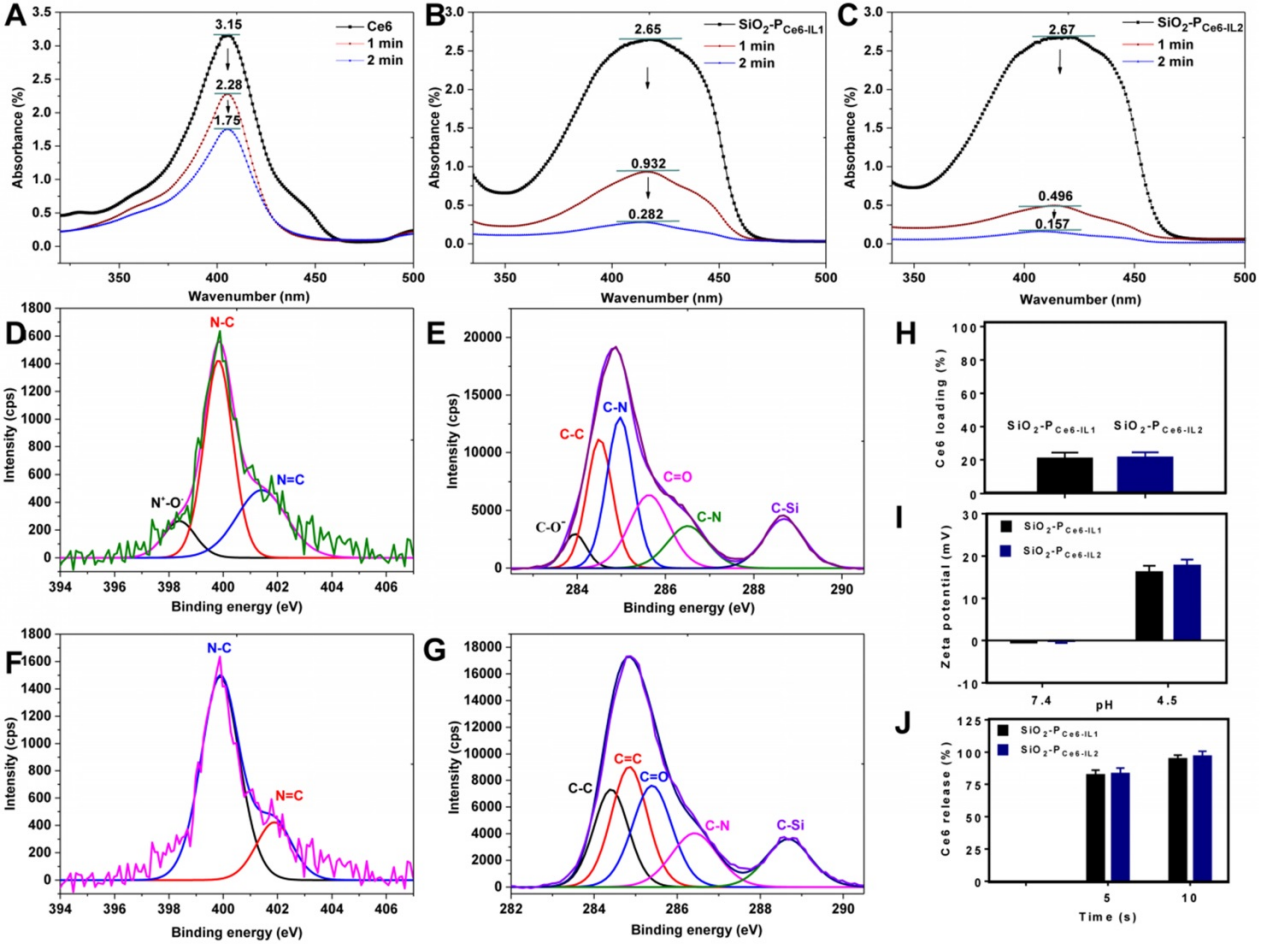
** (A)** The ^1^O_2_ production of Ce6 alone in DMSO. **(B)** The ^1^O_2_ production of SiO_2_-P_Ce6-IL1_ in DMSO. **(C)** The^ 1^O_2_ production of SiO_2_-P_Ce6-IL2_ in DMSO. **(D)** The chemical species of N in SiO_2_-P_Ce6-IL_. **(E)** The chemical species of C in SiO_2_-P_Ce6-IL_. **(F)** The chemical species of N in SiO_2_-P_IL_^+^ when released Ce6 at 24 h. **(G)** The chemical species of C in SiO_2_-P_IL_^+^ when released Ce6 at 24 h. **(H)** Ce6 loading rate of SiO_2_-P_Ce6-IL1_ and SiO_2_-P_Ce6-IL2_. **(I)** Zeta potential of SiO_2_-P_Ce6-IL1_ and SiO_2_-P_Ce6-IL2_ in different pH solution.** (J)** The release rate of Ce6 in pH 4.5 solution.

The production of ^1^O_2_. Because the production of ^1^O_2_ played an important role in PDT, the ^1^O_2_ production of SiO_2_-P_Ce6-IL_ was detected by 1, 3-diphenylisobenzofuran (DPBF). As the ^1^O_2_ can irreversibly oxidize the conjugated structure of DPBF, the reducing absorption band of DPBF corresponded with the ^1^O_2_ generation 38. High ^1^O_2_ generation led to a greater decrease in ultraviolet absorption of DPBF at 410 nm. The DPBF consumption of SiO_2_-P_Ce6-IL_ was greater than that of Ce6 (Figure 3A, B, C). However, the ultraviolet absorption of Ce6 and SiO_2_-P_Ce6-IL_ at 410 nm was not decreased significantly after illumination for 1 and 2 min (Figure S7). The consumption of DPBF was mainly caused by the generation of ^1^O_2_. The high ^1^O_2_ generation efficiency may be caused by polyionic liquids providing a special solvent environment and could improve the stability of the photosensitive structure 39. Due to the short lifetime of ^1^O_2_, the rapid and massive ^1^O_2_ was expected to significantly improve the efficacy of PDT. Compared with SiO_2_-P_Ce6-IL2_, the ^1^O_2_ generation of SiO_2_-P_Ce6-IL1_ was lower (Figure 3B, C). This may also be caused by the entanglement of long-chained P_Ce6-IL1_ with SiO_2_ which prevented ^1^O_2_ from diffusing out of the polymer shell of SiO_2_-P_Ce6-IL1_ and made it undetectable (the shell thickness was 20 nm, and the diffusion distance of ^1^O_2_ was only approximately 10 nm). More ^1^O_2_ production made SiO_2_-P_Ce6-IL2_ have more oxidation capacity to combat biofilm infection.

### The rapid acid responsive ability

The XPS was used to examine the chemical species of cation-anion bond (N^+^-O^-^) and COO^-^ of anion (Ce6) to measure the acid responsive ability of SiO_2_-P_Ce6-IL_. As shown in Figure 3E, F, G and H, the valence peak of the N^+^-O^-^ and C-O^-^ in SiO_2_-P_Ce6-IL_ was disappeared in the acidic solution. As shown in Figure 3J, more than 90% of the Ce6 was released in 10 s. Compared with the traditional acid-sensitive bond, this special ionic bond could significantly increase the release rate of photosensitizer. With the protonation of Ce6, the charge of SiO_2_-P_Ce6-IL_ was inverted and the zeta potentials were changed from -0.3±3.4, -0.6±3.2 mV to +13.8±4.8 and 22.9±3.1 mV, respectively (Figure 3I). Although Ce6-IL was equimolar in SiO_2_-P_Ce6-IL1_ and SiO_2_-P_Ce6-IL2_, the changes in charge were different. Compared with SiO_2_-P_Ce6-IL2_, the charge variation of SiO_2_-P_Ce6-IL1_ was relatively weaker. This may also be caused by the winding of P_Ce6-IL1_ with SiO_2_ which led to some positive charge neutralization with SiO_2_. The lower positive charge weakened the interaction of SiO_2_-P_IL1_^+^ with PNAG and then affected the punching ability of SiO_2_-P_Ce6-IL1_.

### The binding ability of SiO_2_-P_Ce6-IL_ with biofilm

**Figure 4:**
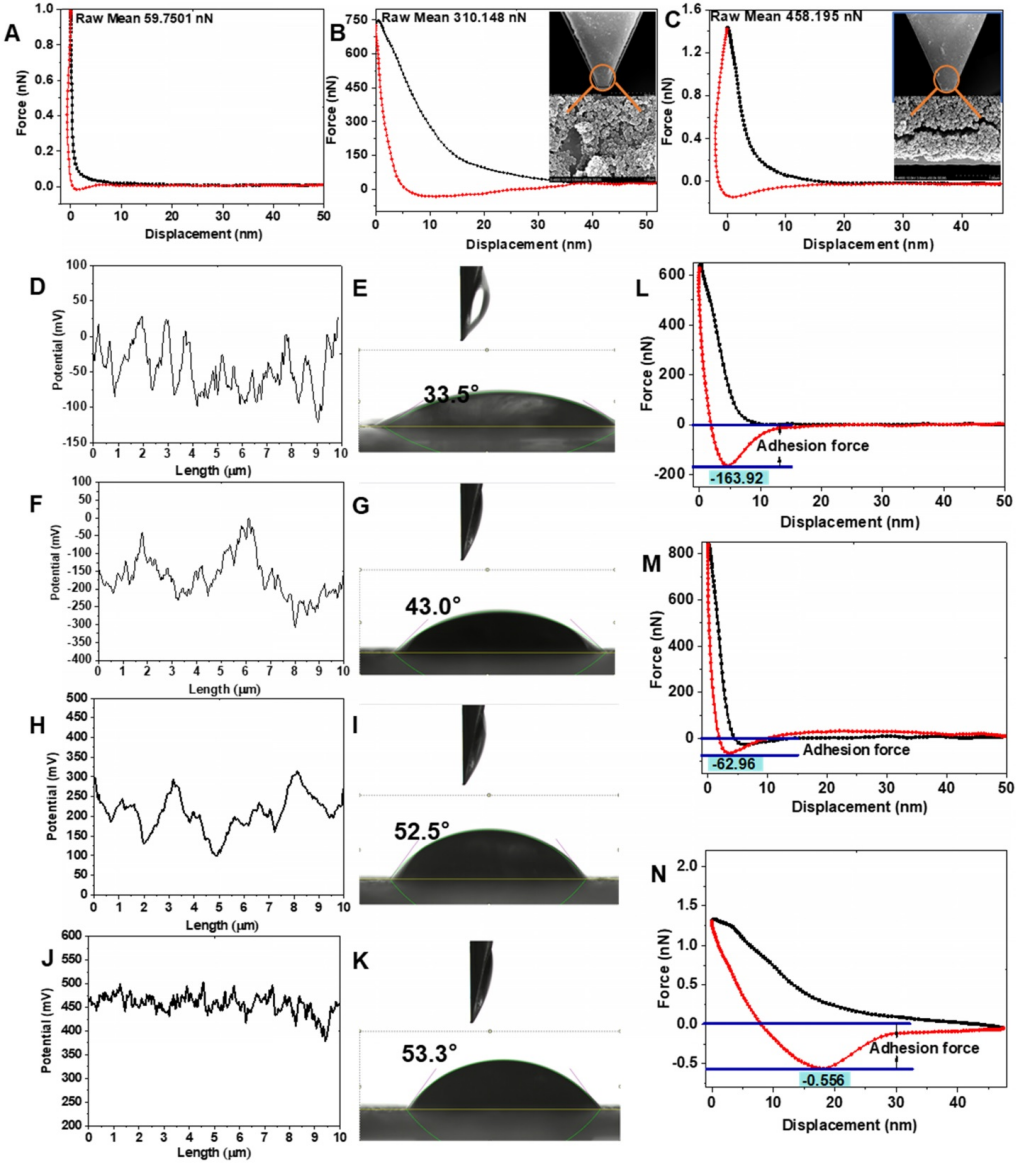
The interaction between biofilm and Ce6 **(A)**, SiO_2_-P_Ce6-IL1_ **(B)**, SiO_2_-P_Ce6-IL2_ **(C)**. **(D)** The surface potential of MRSA. **(E)** The contact angle of MRSA. **(F)** The surface potential of biofilm treated with Ce6. **(G)** The contact angle of biofilm treated with Ce6. **(H)** The surface potential of biofilm treated with SiO_2_-P_Ce6-IL1_. **(I)** The contact angle of biofilm treated with SiO_2_-P_Ce6-IL1_. **(J)** The surface potential of biofilm treated with SiO_2_-P_Ce6-IL2_. **(K)** The contact angle of biofilm treated with SiO_2_-P_Ce6-IL2_. The adhesion force of biofilm treated with Ce6 **(L)**, SiO_2_-P_Ce6-IL1_ **(M)**, SiO_2_-P_Ce6-IL2_ **(N)**.

The SiO_2_-P_IL1_^+^ and SiO_2_-P_IL2_^+^ could bond with negatively charged EPS through electrostatic and hydrophobic interactions. As shown in Figure 4B and C, the interactions of SiO_2_-P_Ce6-IL1_ and SiO_2_-P_Ce6-IL1_ with EPS were 310.15 and 458.20 nN, respectively. However, the interaction of Ce6 with EPS was only 59.75 nN (Figure 4A). The high charged density polyionic liquids with short-chains greatly enhanced the interaction between SiO_2_-P_Ce6-IL2_ and EPS. The stronger interaction provided an opportunity to combine and create holes in the biofilm.

### Biofilm elimination *in vitro*

A semi-quantitative plate assay was used to test the concentration that could eliminate MRSA biofilm. After illumination for 15 min, the MRSA biofilm that was treated with Ce6 was not eliminated at 100 µM or even at 500 µM (Figure S8). Compared with the SiO_2_-P_Ce6-IL1_, SiO_2_-P_Ce6-IL2_ could eliminate MRSA biofilm at 100 µM. To further demonstrate the PDT efficiency of SiO_2_-P_Ce6-IL_, the residual biofilms were dispersed under ultrasonication, and the bacterial viability was analyzed by plate counting. Figure S9 displays the visual images of the agar plates and summarizes the number of bacteria after treating with Ce6 and SiO_2_-P_Ce6-IL_ at 100 µM. The Ce6 alone could not destroy the MRSA bacteria embedded in the biofilm. With the “super-porogen”, the live stationary phase MRSA was significantly disrupted compared with the Ce6 group. Furthermore, the number of MRSA clearly decreased after treating with SiO_2_-P_Ce6-IL_ versus Ce6, particularly for SiO_2_-P_Ce6-IL2_. This demonstrated that the SiO_2_-P_Ce6-IL_ with a short and high charged density chain not only effectively eradicated the biofilm but also inactivated the embedded MRSA.

### The physicochemical properties of biofilm

To study the effect of SiO_2_-P_Ce6-IL_ on the properties of biofilm, the surface potential, hydrophobicity, mechanical and adhesion properties which are key factors in maintaining the structure and protection function of biofilm are examined 40, 41. After adding Ce6, the surface potential of MRSA biofilm decreased from -52 to -101 mV due to the COO^-^ of Ce6 that existed on the biofilm surface (Figure 4F). For the treatment group of SiO_2_-P_Ce6-IL1_ and SiO_2_-P_Ce6-IL2_, the surface potential increased from -42 to 265 and 450 mV, respectively (Figure 4H and J). SiO_2_-P_Ce6-IL2_ had a great influence on the surface potential of MRSA biofilm as a short and high charged density structure of the polyimidazole cation made P_IL2_^+^ repel each other, and it was fully integrated with the biofilm. In addition to the change in surface potential, the hydrophobicity of the MRSA biofilm also increased from the insertion of hydrophobic dodecyl. The contact angles of the MRSA biofilm treated by SiO_2_-P_Ce6-IL1_ and SiO_2_-P_Ce6-IL2_ changed from 33.5° to 52.5° and 53.3°, respectively (Figure 4I and K). The Young's modulus of the MRSA biofilm was 598.12 kpa. After treating with Ce6, SiO_2_-P_Ce6-IL1_ and SiO_2_-P_Ce6-IL2_, the Young's modulus of the biofilm was 435.29, 273.95, and 149.19 kpa, respectively (Figure S10). The mechanical properties after treating with SiO_2_-P_Ce6-IL2_ degraded significantly. The weakened mechanical properties demonstrated that the stronger interaction between SiO_2_-P_Ce6-IL2_ and MRSA biofilm could more effectively destroy the structural integrity of MRSA biofilm. In addition, as the mechanical stability was damaged, the adhesion force of the MRSA biofilm treated by SiO_2_-P_Ce6-IL1_ or SiO_2_-P_Ce6-IL2_ was also decreased from 163.9 nN to 62.96 and 0.556 nN, respectively (Figure 4M and N). Compared with SiO_2_-P_Ce6-IL1_, SiO_2_-P_Ce6-IL2_ almost completely eliminated the adhesion of the MRSA biofilm. This probably means that the short-chained and high-density poly-imidazole cations could efficiently combine with sticky substances, and then quickly eliminated the adhesion. These results demonstrated that SiO_2_-P_Ce6-IL2_ with a short and high density poly-dodecyl-imidazole cations could more effectively damage the physical and chemical properties of MRSA biofilm.

### The hole-making ability

**Figure 5:**
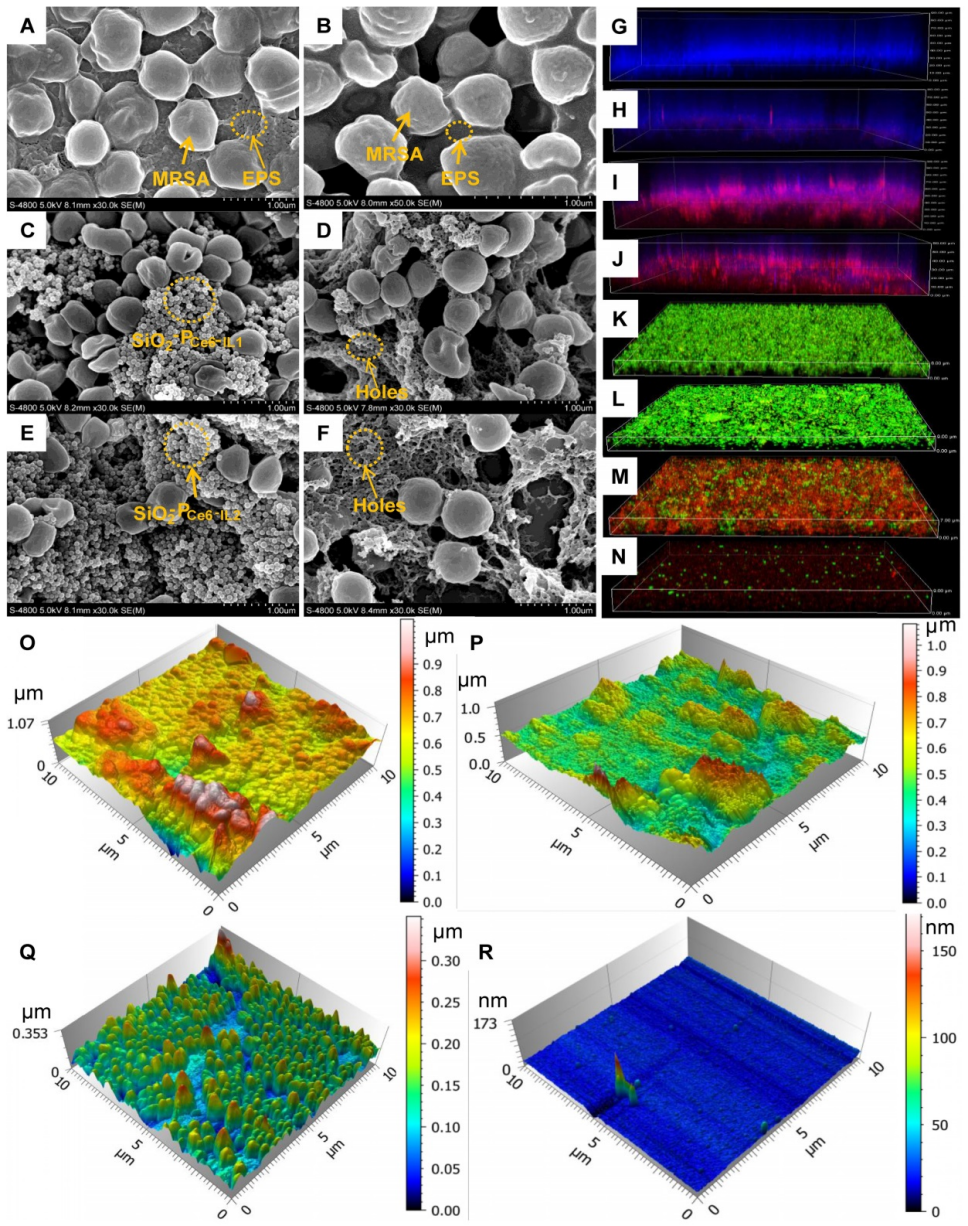
**The morphology of MRSA biofilm. (A)** MRSA biofilm. **(B)** Treated with Ce6 for 10 min. **(C)** Treated with SiO_2_-P_Ce6-IL1_ for 10 min. **(D)** The holes of MRSA biofilm after SiO_2_-P_Ce6-IL1_ removing. **(E)** Treated with SiO_2_-P_Ce6-IL2_ for 10 min. **(F)** The holes of MRSA biofilm after SiO_2_-P_Ce6-IL2_ removing. **(G)** MRSA biofilm (blue). **(H)** The fluorescence imaging (red) of Ce6 in biofilm after treated with Ce6 alone. **(I)** The fluorescence imaging of Ce6 in biofilm after treated with SiO_2_-P_Ce6-IL1_. **(J)** The fluorescence imaging of Ce6 in biofilm after treated with SiO_2_-P_Ce6-IL2_. **(K)** The live (green) & dead (red) bacteria in biofilm. **(L)** The live & dead bacteria in biofilm after treated by Ce6 with illumination for 15 min (5 mW/cm^2^). **(M)** The live &dead bacteria in biofilm after treated by SiO_2_-P_Ce6-IL1_ with illumination for 15 min (5 mW/cm^2^). **(N)** The live&dead bacteria in biofilm after treated by SiO_2_-P_Ce6-IL2_ with illumination for 15 min (5 mW/cm^2^). **(O)** The morphology of MRSA biofilm. **(P)** The morphology of MRSA biofilm treated by Ce6 with illumination for 15 min (5 mW/cm^2^). **(Q)** The morphology of MRSA biofilm treated by SiO_2_-P_Ce6-IL1_ with illumination for 15 min (5 mW/cm^2^). **(R)** The morphology of MRSA biofilm treated by SiO_2_-P_Ce6-I2_ with illumination for 15min (5 mW/cm^2^).

The surface potential, hydrophobicity, mechanical and adhesion force must be changed to destroy the structural integrity of the biofilm. As shown in Figure 5ⅰ, the MRSA biofilm composed EPS and incorporated MRSA bacteria. For the charge reversal, SiO_2_-P_IL1_^+^ and SiO_2_-P_IL2_^+^ could firmly adsorb on the EPS (Figure 5C and E). After interaction for 10 min, many holes in the EPS were actually observed by SEM (Figure 5D and F), especially for SiO_2_-P_Ce6-IL2_.

### Location and ROS of Ce6 in the biofilm

The biofilms as a natural barrier prevent the photosensitizer from entering. After the SiO_2_-P_Ce6-IL_ treatment, Ce6 could easily enter into the biofilm through holes and concentrate in the biofilm. The location of Ce6 was confirmed by a CLSM. As shown in Figure 5G, the MRSA biofilm exhibited integrity (blue) and it was difficult for Ce6 alone enter (Figure 5H). However, the blue fluorescence intensity of the MRSA biofilm treated by SiO_2_-P_Ce6-IL1_ and SiO_2_-P_Ce6-IL2_ was decreased, and a high concentration of Ce6 was detected in the MRSA biofilm (Figure 5I and J). More importantly, the high concentration of ROS after treating with SiO_2_-P_Ce6-IL_ was observed in the biofilm (Figure S11). Because of the effective accumulation of ROS in the biofilm, almost all of the MRSA bacteria were killed by SiO_2_-P_Ce6-IL2_ (Figure 5M and N).

### The morphology of the MRSA biofilm

The MRSA biofilm treated with Ce6, SiO_2_-P_Ce6-IL1_ or SiO_2_-P_Ce6-IL2_ with illumination was examined by AFM and SEM. As shown in Figure 5B and P, the treatment of Ce6 alone had almost no effect on the MRSA embedded in biofilm other than a slight influence on the surface structure of the biofilm. However, the structure and morphology MRSA biofilm was destroyed after treated by SiO_2_-P_Ce6-IL1_ or SiO_2_-P_Ce6-IL2_ with illumination for 15 min, especially for SiO_2_-P_Ce6-IL2_ (Figure 5Q, R and Figure S12).

### Anti-biofilm activity *in vivo*

**Figure 6:**
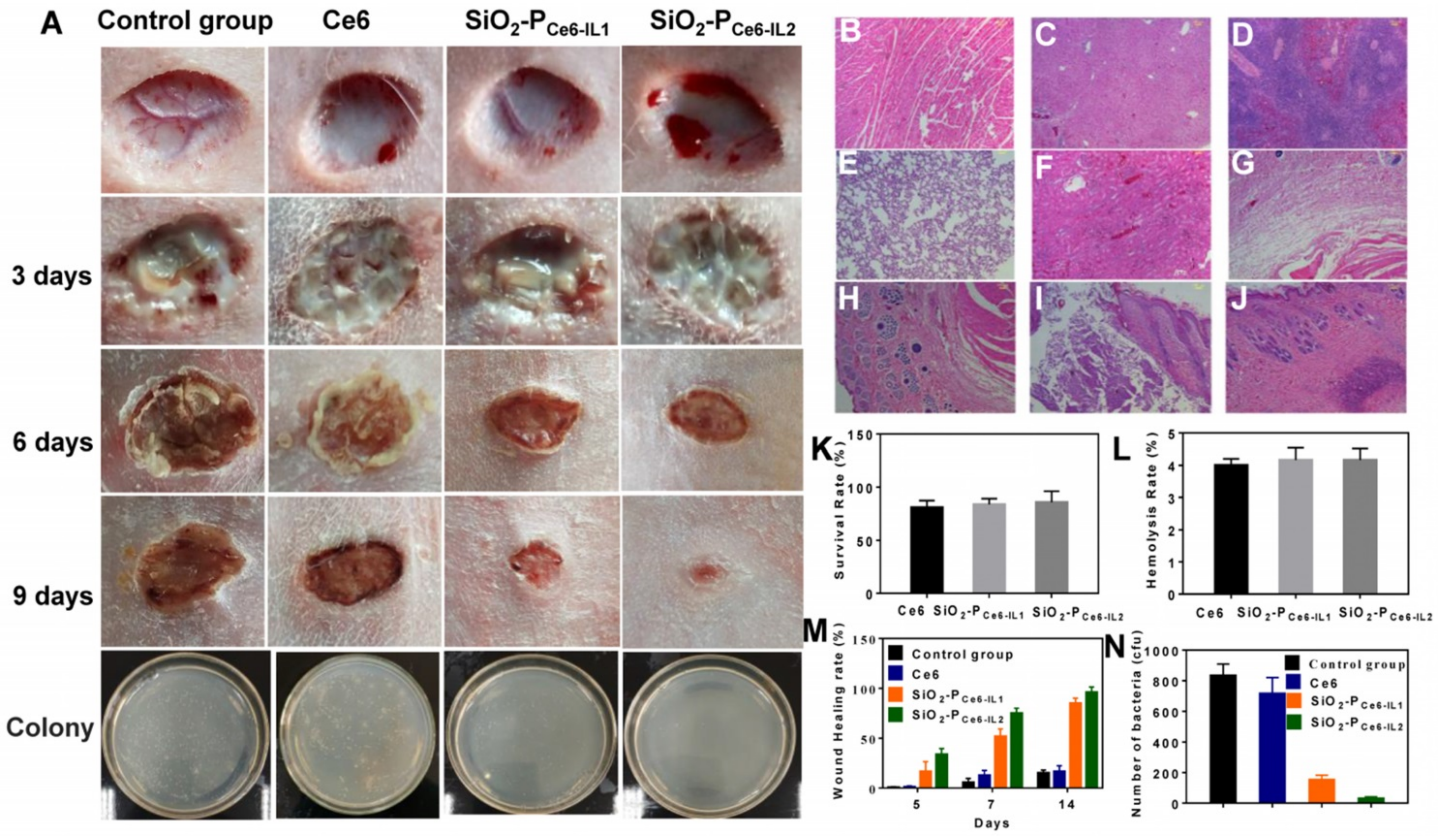
** Antibacterial effect *in vivo* and biocompatibility assay*.* (A)** Photomicroscope images of wounds at different days. H&E staining. **(B)** Heart. **(C)** Liver. **(D)** Spleen. **(E)** Lung. **(F)** Kidney. **(G)** Subcutaneous.** (H)** Wound areas. **(I)** Infected skin tissue. **(J)** Infected skin treated with SiO_2_-P_Ce6-IL2_. **(K)** Cytotoxicity. **(L)** Hemolysis rate. **(M)** Wound healing rate. **(N)** The number of bacterial colony-forming units obtained from control, and after treated by Ce6, SiO_2_-P_Ce6-IL1_, SiO_2_-P_Ce6-IL2_ with illumination for 15 min (50 µL of Ce6, SiO_2_-P_Ce6-IL_ and SiO_2_-P_Ce6-IL2_ (containing 100 µM Ce6), 5 mW/cm^2^).

To assess the anti-biofilm activity *in vivo*, the cutting model was fabricated on the back of rabbit. The wounds were injected with 50 μL of 10^8^ cfu/mL MRSA to construct the MRSA biofilm infection model. The infected rabbits were divided into four groups: treated by PBS, Ce6, SiO_2_-P_Ce6-IL1_ and SiO_2_-P_Ce6-IL2_ with illumination for 15 min. Figure 6 showed the photographs of the wounds in 1-9 days. All the infected groups showed certain degree of pyosis in 3 days. 50 µL of PBS, Ce6, SiO_2_-P_Ce6-IL_ (Ce6 100 µM) was dropped on the wound area at the corresponding groups and irradiated with 660 nm light (5 mW/cm^2^) for 15 min. In 6 days, the pyosis of infected wounds that treated with SiO_2_-P_Ce6-IL1_ and SiO_2_-P_Ce6-IL2_ disappeared. In 9 days, the group of SiO_2_-P_Ce6-IL2_ showed better healing than other groups (Figure 6M). To assess the bactericidal effect on the wounds, the MRSA on the wounds at 14 days were cultured, and then colonies were counted. For only few colonies formed after incubating for 24 h (Figure 6N), the SiO_2_-P_Ce6-IL2_ exhibited the remarkable therapeutic effect for combating MRSA biofilm infection.

### Biocompatibility assay

As SiO_2_-P_Ce6-IL_ was in direct contact with tissues and blood in practical clinical applications, the biocompatibility was evaluated. The cytotoxicity and hemolysis rate of the SiO_2_-P_Ce6-IL1_ and SiO_2_-P_Ce6-IL2_ at 100 µM were above 90% and less than 5%, respectively (Figure 6K and L). In addition, to evaluate the safety of the SiO_2_-P_Ce6-IL2_, the heart, liver, spleen, lung, kidney and embedded tissue were also harvested for H&E staining. The pathological and histopathological studies showed that SiO_2_-P_Ce6-IL_ could not cause the damage to embedded tissue and major organs. The SiO_2_-P_Ce6-IL_ could be as a safe material against biofilm infection.

## Discussion

In summary, we reported a novel antibacterial system to solve the release and transport barrier problem of photosensitizers. Compared with the traditional system, the SiO_2_-P_Ce6-IL_ could rapidly concentrate the photosensitizers in biofilm and control infection at the early stages. The *in vitro* and *in vivo* results indicated that SiO_2_-P_Ce6-IL_ can effectively reduce the inflammatory stage of the wound and accelerate wound healing. The biocompatibility results indicated that SiO_2_-P_Ce6-IL_ could be an effective and safe therapeutic method for controlling MRSA biofilm infection. Furthermore, the highly efficient utilization of photosensitizers could reduce economic losses. As Ce6 has an excellent bactericidal effect on Gram-positive bacteria, the SiO_2_-P_Ce6-IL_ is expected to be an effective strategy for other positive bacterial biofilm infections in clinical applications.

## Materials and Methods

### Materials

1, 3-diphenylisobenzofuran (DPBF) and 3-(4, 5-dimethyl thiazol-2-yl)-2, 5-diphenyl-tetrazolium bromide (MTT) were purchased from Sigma-Aldrich. Ce6 was purchased from Frontier Scientific. KOH, Triton x-100, methanol, absolute ethanol, cyclohexane, hexanol, and triethylamine were purchased from Sinopharm Chemical Reagent Co., Ltd. Crystal violet, N, N, N', N'', N''-pentamethyldiethylenetriamine, CuCl, 2-bromoisobutyryl bromide, tetraethyl orthosilicate (TEOS), ammonia solution, and 3-aminopropyl triethoxysilane (APTES) were purchased from Aladdin. 1-vinyl-3-dodecyl imidazole bromide (IL) was kindly provided by the Key Laboratory of Space Applications Physics and Chemistry, Northwestern Polytechnical University. Methicillin-resistant Staphylococcus aureus (MRSA ATCC 33591) was provided by Xijing Hospital (The resistant criterion of MRSA to the drugs as follows: the MIC of OX, AK and EM was 0.486, 0.489 and 0.491 mg/mL, respectively; the MIC of CL and CIP was 0.015 and 0.063 mg/mL, respectively). LB-medium and agar were purchased from MP Biomedicals. Twenty-four pore plate circular cell crawling slices were purchased from WHB (WHB-24-CS, China). Alexa Fluor 647 was purchased from Thermo Fisher Scientific. Cellular ROS Assay Kit (deep red) ab 186029 was purchased from abcam.

### The interaction of cation and PNGA

The binding energy (Δ*E*) of 1-vinyl-3-dodecyl imidazole and poly 1-vinyl-3-dodecyl imidazole (1 unit) to PNGA was calculated using Materials Studio. The simulation parameters were as follows: Forcite (module), universal (forcefield), current (charge), fine (quality), atom-based (electrostatic), van der Waals, cubic spline (truncation) cutoff distance of 12.5 Å, spline of 1 Å, and buffer width of 0.5 Å.

### Synthesis and characterization of 1-vinyl-3-dodecyl imidazole Ce6 (Ce6-IL)

The cation 1-vinyl-3-dodecyl imidazole bromide (IL) and anion Ce6 were assembled into Ce6-IL by an anion exchange reaction. The characterization of Ce6-IL was reported in our previous reports 35. The pKa values of the carboxylic acid groups of Ce6-IL have been determined by titration with NaOH according to the references 42. The pH of the solution was measured using a calibrated glass electrode on a pH meter (M-T FE28, Switzerland) at 25°C.

### Preparation of SiO_2_-Br

SiO_2_ nanoparticles were synthetized by three phase emulsion polymerization. In briefly, 38.0 mL of cyclohexane, 12.0 mL of Triton x-100, 8.0 mL of hexanol and 2.0 mL of distilled water were added to a flask and stirred for 30 min at 1100 rpm; then 500 μL of TEOS and 1.8 mL of ammonia (25%) were added for a reaction for 24 h at room temperature. The obtained SiO_2_ was washed several times by distilled water, ethanol and dried by vacuum freeze-drying. To study the influence of chain length and charge density on antibacterial properties, Br of two different densities was grafted onto the surface of SiO_2_. First, 50 mg of SiO_2_ was dispersed in anhydrous ethanol, and then 60 µL of APTES was added for a reaction for 48 h at 70 ℃. After washing with alcohol and water three times, SiO_2_-NH_2_ was obtained. Second, the SiO_2_-NH_2_ dissolved into anhydrous acetonitrile and 0.2 mL of 2-bromoisobutyryl bromide, 0.4 mL of anhydrous three ethylamine or 1.0 mL of 2-bromoisobutyryl bromide, and 2.0 mL of anhydrous three ethylamine were added for a reaction for 12 h in an ice bath. After the reaction, the two Br of different densities was washed by ethanol. The percentage of Br was examined by EDX.

### Preparation and characterization of SiO_2_-P_Ce6-IL_

Poly Ce6-IL of different chain lengths and charge densities were grafted onto SiO_2_ by ATRP. In briefly, SiO_2_-Br of two different densities were dissolved in 85% ethanol solution. After ultrasonic dispersion, 50 mg of Ce6-IL, 200 μL of PMDTA and 30 mg of CuCl were added into a flask for a reaction for 6 h under nitrogen protection. The morphology of SiO_2_-P_Ce6-IL_ was examined by TEM and SEM. The zeta potential of SiO_2_-P_IL1_^+^ and SiO_2_-P_IL2_^+^ in solutions with a pH of 7.4 and 4.5 were measured at 25 °C by a Delsa Nano C particle analyzer (Beckman Coulter Ireland Inc.).

### The loading and release rate of Ce6

1.0 mg of SiO_2_-P_Ce6-IL_ was dispersed in a solution with a pH of 7.4. The absorbency of Ce6 at 660 nm was examined by UV-Vis (MAPADA, China). The Ce6 loading rate was eventually calculated by loading Ce6/carrier weight×100%. After the two kinds of SiO_2_-P_Ce6-IL_ were dispersed in a solution with a pH of 4.5, centrifugate was collected at 5 and 10 s by ultrafiltration. Then, the absorbency of Ce6 in centrifugate was examined, and the release rate was calculated.

### The generation of ROS and ^1^O_2_ assay

DPBF was used as a probe to measure the generation of ^1^O_2_ according to the literature 43. DMSO solution (2.0 mL) containing SiO_2_-P_Ce6-IL_ (Ce6 100 μM) and DPBF (100 μM) was irradiated by 660 nm light (5 mW/cm^2^). The absorbance of DPBF at 410 nm was recorded when illuminated for 1 and 2 min. As the control group, the ultraviolet absorption of Ce6 and SiO_2_-P_Ce6-IL_ at 410 nm without DPBF was detected after illumination for 1 and 2 min. ROS generation in living cells could detect using Cellular ROS Assay or DCFH-DA 44, 45. In this work, the ROS of Ce6 in the MRSA biofilm wan detected by Cellular ROS Assay (deep red). After the Ce6, SiO_2_-P_Ce6-IL1_ and SiO_2_-P_Ce6-IL1_ (Ce6 100 μM) solutions combined with MRSA biofilm 10 min, Cellular ROS Assay was added. The ROS was produced with the 660 nm laser irradiation. The fluorescence imaging of ROS was evaluated by confocal laser scanning microscopy (CLSM). The excitation and emission wavelengths were 650 and 675 nm, respectively.

### Culturing MRSA biofilm

The cell crawling slices and 2.0 mL of MRSA (1×10^7^ cfu/mL) in LB medium (2% sucrose) with a pH of 4.5 were placed into a 24 pore-plate and MRSA biofilm was cultured at 37 °C for 72 h. After the LB medium was removed, the MRSA biofilm attached on the slices was harvested. According to the literature 46, the characterization of MRSA was as follows: the adhesion was 163.92 nN, the thickness was 90 µM, and the contact angle of the surface was 33.5° (hydrophilic).

### Biofilm elimination* in vitro*

The elimination of MRSA biofilm was examined by semi-quantitative determination with crystal violet staining 47. First, the obtained MRSA biofilm was rinsed briefly by PBS to remove planktonic bacteria. Afterward, 20 μL of different concentrations of Ce6 and SiO_2_-P_Ce6-IL_ were added into 96-well plates to interact with the biofilm for 10 s, and then illuminated for 15 min (5 mW/cm^2^). Second, the residual biofilm was stained with 200 μL of 1.0% crystal violet solution for 30 min, and 200 μL of ethanol was added to dissolve the crystal violet. The concentrations of Ce6 and SiO_2_-P_Ce6-IL_ ranged from 0 to 500 μM (0, 0.01, 0.05, 0.1, 1.0, 50, 100 and 500 μM).

### Photodynamic inactivation of biofilm

MRSA biofilm was cultured for 72 h in 96-well plates and washed with PBS three times. Then, 50 µL of PBS, Ce6 and two kinds of SiO_2_-P_Ce6-IL_ (Ce6 100 µM) were added into each well and allowed to interact with biofilm for 10 min. Afterward, the 96-well plates were subjected to 660 nm irradiation for 15 min (5 mW/cm^2^). To quantify the viable bacteria, the residual biofilm was detached via low-energy sonication to obtain bacterial suspensions in 1.0 mL of PBS. Then the serially diluted bacteria suspensions were plated on LB agar incubation at 37 °C for 24 h, and the colonies forming units were counted.

### Interaction of SiO_2_-P_Ce6-IL_ with biofilm

The interactions between Ce6, SiO_2_-P_Ce6-IL_ and MRSA biofilm were measured by AFM. A common protocol was employed for the attachment of SiO_2_-P_Ce6-IL1_, SiO_2_-P_Ce6-IL2_ or Ce6 to the AFM tip: the AFM tip (NP-O10, Bruker) was placed in epoxy, which was allowed to cure for some time (total 5 min) 48, 1 µL of SiO_2_-P_Ce6-IL1_, SiO_2_-P_Ce6-IL2_ and Ce6 (120 µM) were placed on the AFM tip. After drying at 80 ℃ for 24 h, the decorated tip was washed by distilled water to eliminate unattached nanoparticles. SEM was used to examine the SiO_2_-P_Ce6-IL_ nanoparticles that terminated on the AFM tip.

### The influence of SiO_2_-P_Ce6-IL_ on biofilm

The changes in the physicochemical properties of the MRSA biofilm were examined by AFM (Dimension FastScan and Dimension Icon, Bruker, Germany) and a contact angle measuring instrument (OCA200, Dataphysics, Germany). The detailed processes were as follows: MRSA biofilm was treated with PBS, 100 µM of Ce6 and SiO_2_-P_Ce6-IL_. After interaction for 10 min, the surface potential, contact angle, mechanical and adhesion properties of MRSA biofilm were examined. The Young's modulus of the MRSA biofilm was examined by Nano Indenter (Piuma, Optics11, Holland).

### Analysis of the hole-forming ability and morphology of the biofilm

To study the hole-forming ability, three pieces of MRSA biofilm treated with 100 µM of Ce6 and SiO_2_-P_Ce6-IL_ without illumination were examined. After interaction for 10 min, the three pieces of MRSA biofilm were washed several times with PBS to remove Ce6 and SiO_2_-P_Ce6-IL_. The effect of superporogen and PDT on the morphology of MRAS biofilm was also examined using three pieces of MRSA biofilm treated with 100 µM of Ce6 and SiO_2_-P_Ce6-IL1_ with illumination for 15 min. AFM and SEM were used to examine the changes in the biofilm.

### Location of photosensitizer

The biofilm was stained and examined with confocal scanning laser microscopy (CSLM) 49. In briefly, 1.0 µM of Alexa Fluor 647-labeled dextran conjugate (molecular weight 10,000; absorbance wavelength 647 nm; emission wavelength 668 nm) was added to culture for 6 h. Then, 20 µL of SiO_2_-P_Ce6-IL1_, SiO_2_-P_Ce6-IL2_ or Ce6 was added to the biofilm to interact for 10 min, after which the biofilm was washed with PBS 3 times to remove residual SiO_2_-P_Ce6-IL_ or Ce6. The CSLM imaging was performed using a Leica TCS SP1 microscope (Leica TCS SP8 STED 3X Super-resolution Confocal Microscope, Germany) equipped with argon ion and helium-neon lasers set at 400 and 640 nm, respectively.

### Animal studies

Healthy New Zealand white rabbits were used in the animal study. The MRSA infected wounds were prepared on the backs of the rabbits. In briefly, 8 week-old rabbits (1.5-1.8 kg) were purchased from the Laboratory Animal Center of the Fourth Military Medical University and divided into four groups: PBS, Ce6, SiO_2_-P_Ce6-IL1_ and SiO_2_-P_Ce6-IL2_ (three rabbits in each group). The rabbits were anesthetized by 2% sodium pentobarbital. After shaving and disinfecting with alcohol, the wounds (d=2 cm) were obtained by surgical procedure on the backs of the rabbits. The infected wounds were treated by a 50 μL of 10^8^ cfu/mL MRSA suspension. When a biofilm-infected wound was observed, the 50 µL or 100 µM of Ce6 or SiO_2_-P_Ce6-IL_ was dropped on the wound area for the corresponding groups. The wounds treated by PBS served as the control group. After 10 min, the infected wound area was irradiated for 15 min (660 nm, 5 mW/cm^2^). The wounds were photographed to observe the healing rate of the wound. To check the antibacterial activity* in vivo*, the bacterial samples treated with PBS, Ce6, SiO_2_-P_Ce6-IL1_ or SiO_2_-P_Ce6-IL2_ at 14 days were collected from the wound area by sterile swab. After culturing for 8 h and diluting 1,000 times, 10 μL of bacterial suspension was spread on the agar culture plate and incubated at 37 ℃ for 24 h to count the number of colonies.

### Biocompatibility assay

The cell viability was evaluated by MTT assays. Normal L929 fibroblast cells were seeded into 96-well plates (6000 cells per well) with 200 μL of DMEM culturing medium in each well for 24 h. Ce6, SiO_2_-P_Ce6-IL1_ or SiO_2_-P_Ce6-IL2_ with concentrations of 100 µM were added to the cells and irradiated by 660 nm light for 15 min (5 mW/cm^2^). After incubation for 48 h, 20 μL of MTT solution (0.1 mg/mL) was added to each well for another 4 h culturing. Then, the medium was removed, and 150 µL of DMSO was added to each well to dissolve the obtained crystals. The absorbance was recorded at 570 nm by a microplate reader (model 550 BioRad).

Fresh blood (3.0 mL) was obtained from the New Zealand white rabbit. After the red cells were diluted to 2% in PBS, Ce6 or SiO_2_-P_Ce6-IL_ (100 µM) was immersed into a tube (5.0 mL for each tube) and incubated at 37 ℃ for 3 h. The red cells were centrifuged and the supernatant containing hemoglobin was detected using UV-Vis. The OD values were recorded at 540 nm. The red cells with water and PBS were the positive control and negative control, respectively. The hemolysis rate was determined by the following equation 50-52:


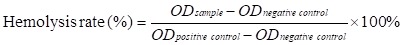


The positive control and the negative control were water and normal saline, respectively.

SiO_2_-P_Ce6-IL2_ was implanted subcutaneously for 4 weeks. After the experiment, the embedded tissue, wound tissue, heart, liver and spleen, lungs and kidneys were harvested to study the biocompatibility by H&E staining analysis.

All experimental animal operating procedures were in line with the laboratory animal care and usage guidelines.

All data were expressed as the means ± SD. Differences between groups were examined for significance with a two-tailed Student's test and significance was set at p < 0.05.

## Figures and Tables

**Table 1:** The energy and the binding energy (Δ*E*) of P_IL_^+^ with PNAG

	E (Kcal·mol^-1^)	ΔE (Kcal·mol^-1^)
IL^+^ P_IL_^+^	51.210512 135.791181	
PNAG	112.303170	
IL^+^ with PNAG	156.674847	-6.839135 ^[A]^
P_IL_^+^ with PNAG	236.882540	-23.442899 ^[B]^

[A] Δ*E*_1_ = E_IL_^+^_-PNAG_ - E_IL_^+^- E_PNAG._ [B] Δ*E*_2_ = E_PIL_^+^_-PNAG_ - E_PIL_^+^- E_PNAG._
