# Supplementary material for: pH responsive superporogen combined with PDT based on poly Ce6 ionic liquid grafted on SiO2 for combating MRSA biofilm infection
Source: Theranostics. 2020 Mar 26;10(11):4795–808. doi: 10.7150/thno.42922 (PMC7163436; doi:10.7150/thno.42922)
Supplement: Supplementary file 1 — Supplementary figures. [file thnov10p4795s1.pdf]

Supporting Information

pH responsive superporogen combined with PDT based on poly Ce6 ionic liquid grafted on SiO_2 for combating MRSA biofilm infection

Chaoli Wang [a*], Peng Chen [a*], Youbei Qiao [a*], Yuan Kang [b], Chaoren Yan[b], Zhe Yu [a], Jian Wang [c], Xin He[a], and Hong Wu[a✉]

[a] Department of pharmacy, Air Force Medical University, Xi'an, 710032, Shaanxi Province, China

[b] Key Laboratory of space applications physics and chemistry, Northwestern Polytechnical University, Xi'an, 710032, Shaanxi Province, China

[c] Department of Thoracic Surgery, Tangdu Hospital, Air Force Medical University, Xi'an, 710032, Shaanxi Province, China

[*] These authors contributed equally to this work

✉Corresponding author: Hong Wu (wuhong@fmmu.edu.cn). Department of pharmacy, Air Force Medical University, Xi'an, 710032, Shaanxi Province, China

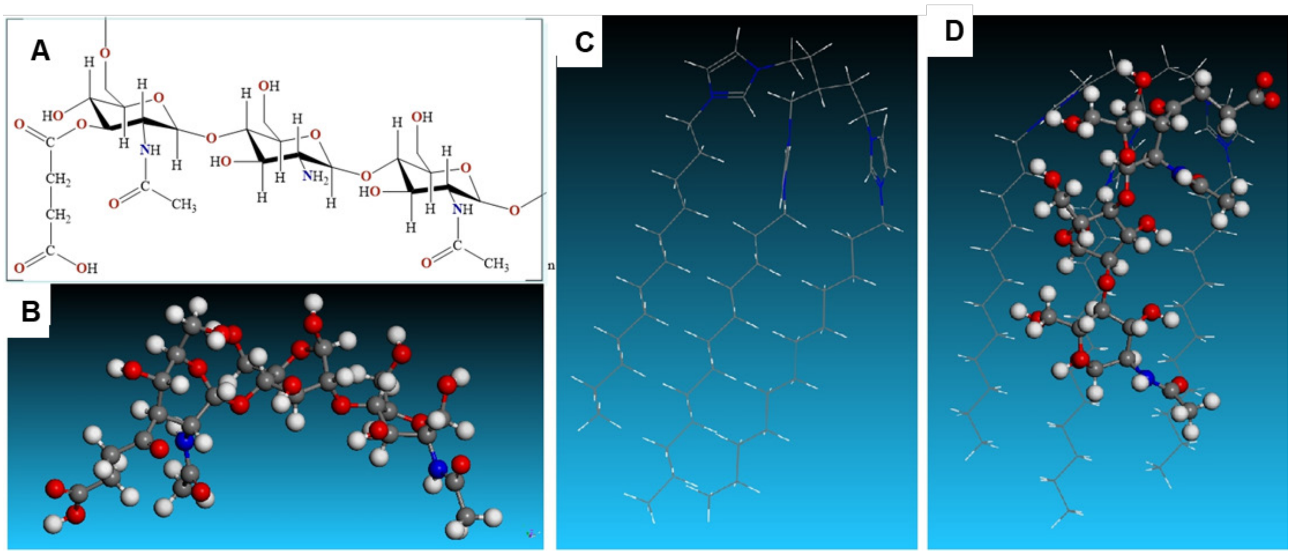

Figure S1. The structure and characterization of PNAG and P_{IL}^{+}

(A) The structure of PNAG. (B) The optimized structure of PNAG.

(C) The optimized structure of P_{IL}^{+}. (D) The optimized structure of PNAG and P_{IL}^{+} complex.

The simulation parameters was as follows: Forcite (module), universal (forcefield), current (charge), fine (quality), atom based (electrostatic), van der Waals, cubic spline (truncation) cutoff distance 12.5 Å, spline 1 Å, and buffer width 0.5 Å.

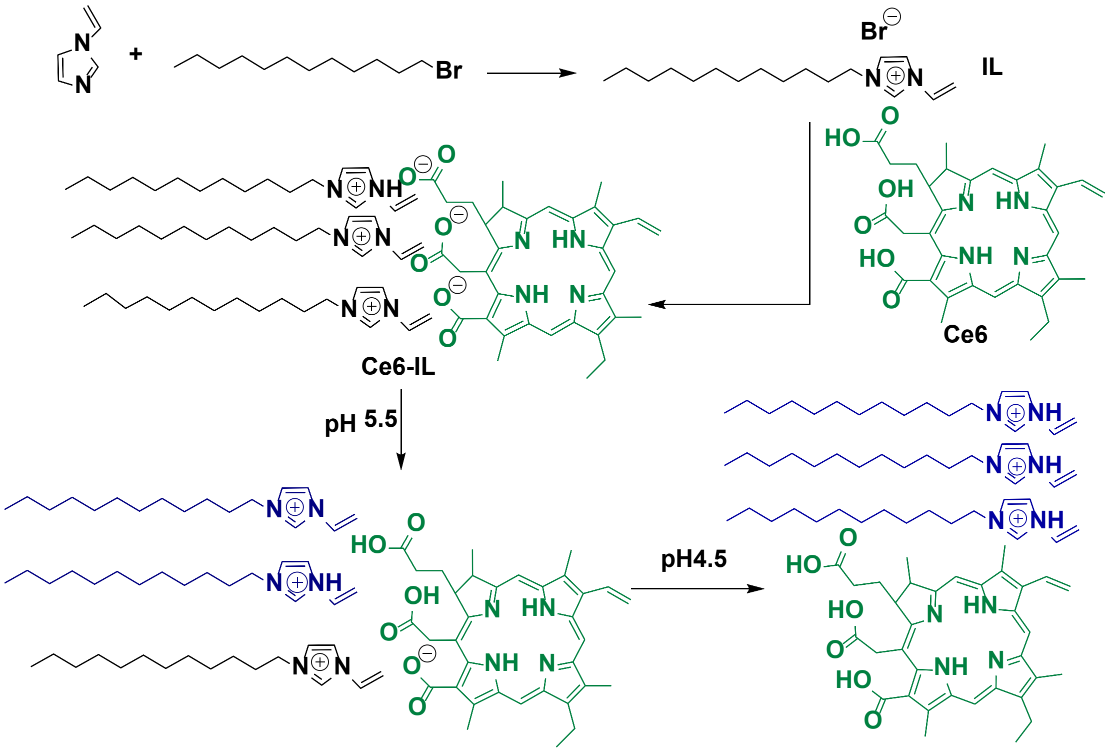

Figure S2. Schematic illustration of the synthetic route of Ce6-IL

The cation 1-vinyl-3-dodecyl imidazole (IL) and anion Ce6 were assembled into Ce6-IL by an anion exchange reaction.

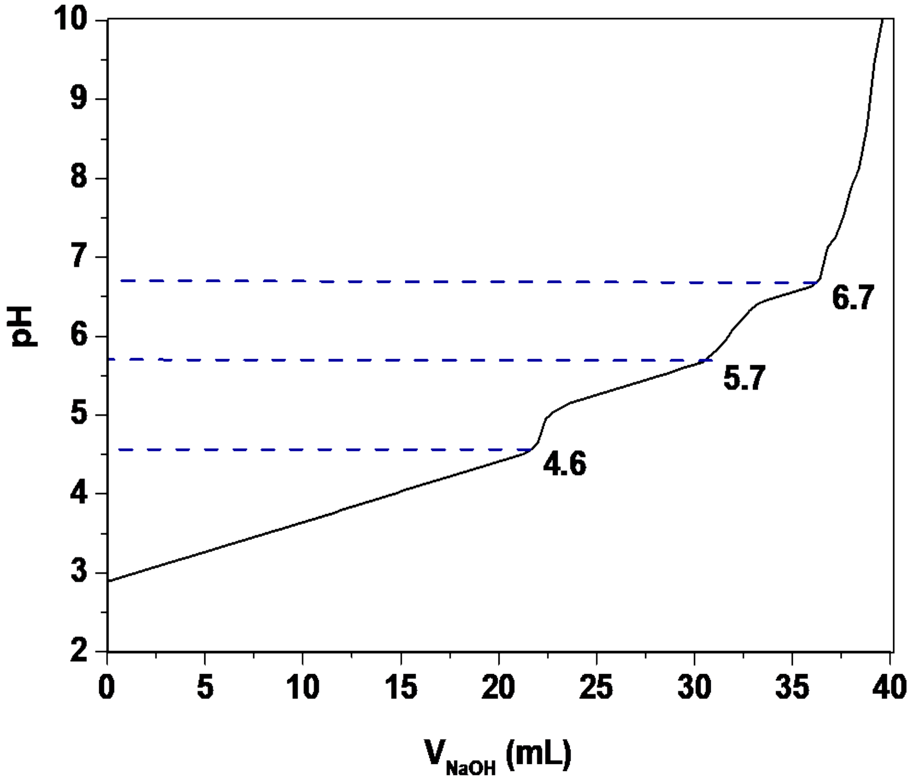

Figure S3. The pKa of Ce6-IL

The pKa values of the carboxylic acid groups of Ce6-IL was determined by titration with NaOH. As showed in the Figure S3, the pKa of Ce6 was 4.6, 5.7, and 6.7, respectively.

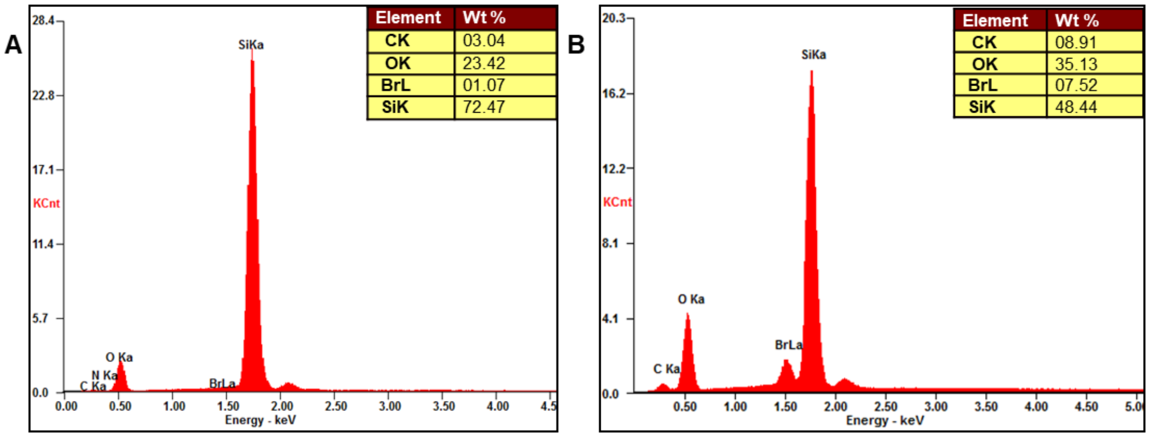

Figure S4. The element analysis

(A) SiO_2-Br_1. (B) SiO_2-Br_2.

The SiO_2-NH_2 dissolved into anhydrous acetonitrile and 0.2 mL of 2-bromoisobutyryl bromide, 0.4 mL of anhydrous three ethylamine or 1.0 mL of 2-bromoisobutyryl bromide, 2.0 mL of anhydrous three ethylamine were added for reaction 12 h in an ice bath. After reaction, two densities of Br were washed by ethanol three times. The EDX result showed that the percentage of Br on the SiO_2 was 1.07% and 7.52%, respectively.

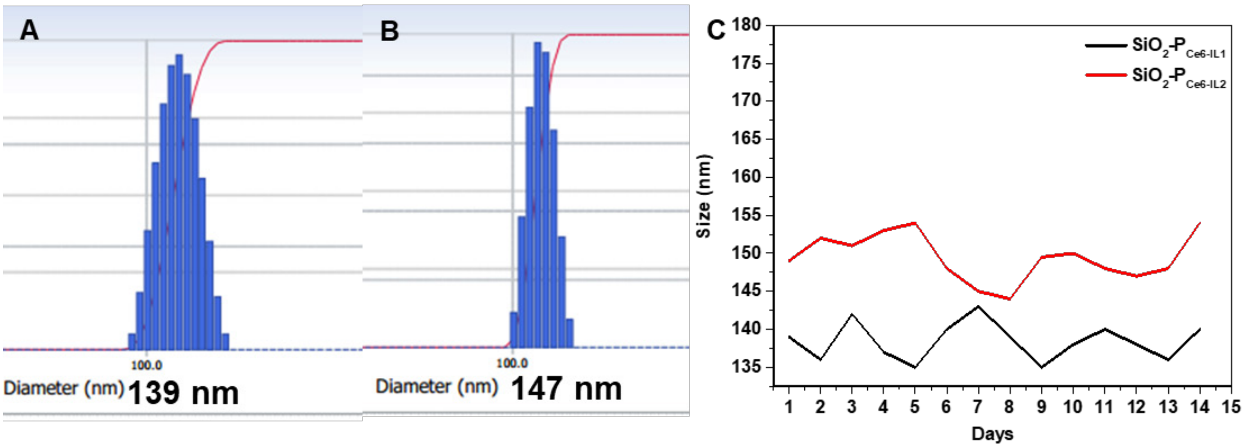

Figure S5. The DLS of SiO_2-$P_{Ce6\text{-}IL1}$ and SiO_2-$P_{Ce6\text{-}IL2}$

(A) The hydration radius of the SiO_2-$P_{Ce6\text{-}IL1}$. (B) The hydration radius of the SiO_2-$P_{Ce6\text{-}IL1}$ and SiO_2-$P_{Ce6\text{-}IL2}$. (C) The stability of SiO_2-$P_{Ce6\text{-}IL}$.

As showed in Figure S8, the hydration radius of the SiO_2-$P_{Ce6\text{-}IL1}$ and SiO_2-$P_{Ce6\text{-}IL2}$ was 139 and 147 nm, respectively. The dynamic light scattering (DLS) results showed that the SiO_2-$P_{Ce6\text{-}IL}$ has excellent stability in PBS.

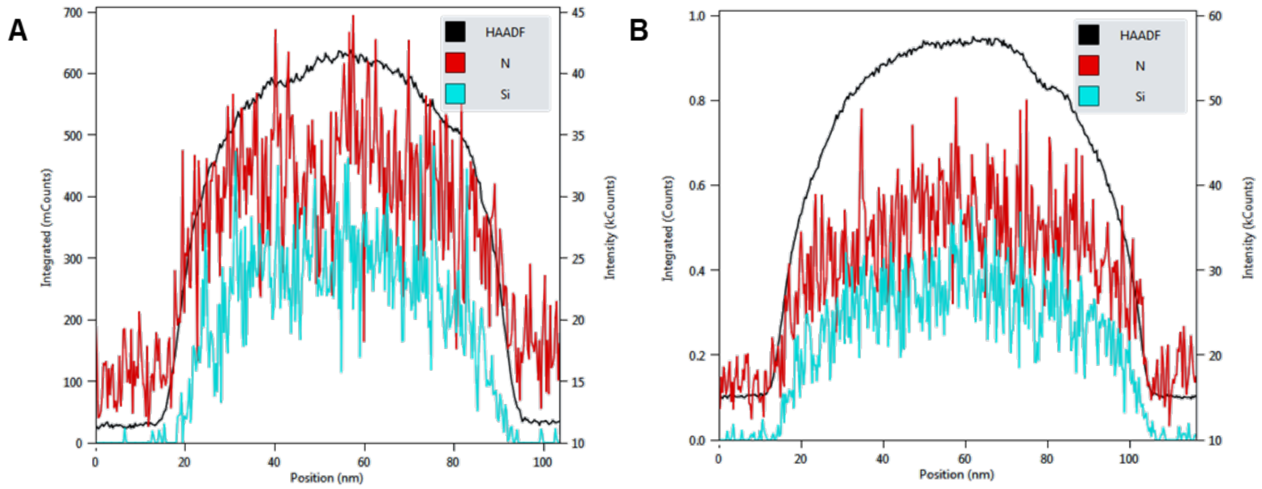

Figure S6. The Microscope (ACTEM) analysis

(A) ACTEM analysis of SiO_2-$P_{Ce6\text{-}IL1}$. (b) ACTEM analysis of SiO_2-$P_{Ce6\text{-}IL2}$.

The location of Si and N was further analyzed by spherical aberration corrected transmission electron microscope (ACTEM). The result showed that the N was on the surface of SiO_2.

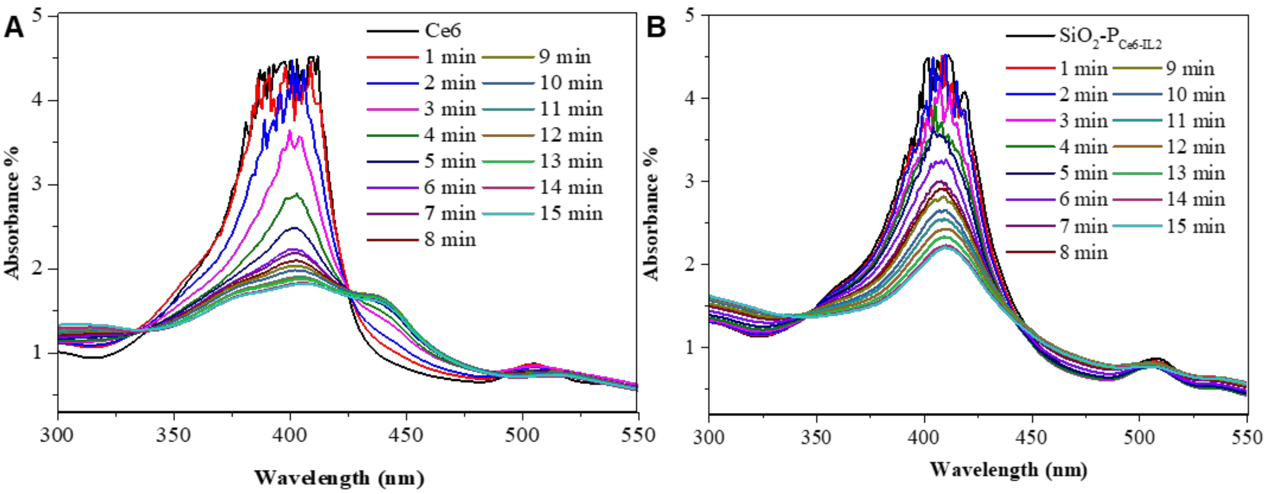

Figure S7. The ultraviolet absorption

(A) The ultraviolet absorption of Ce6 at 410 nm. (B) The ultraviolet absorption of SiO_2-$P_{Ce6\text{-}IL2}$ at 410 nm.

The ultraviolet absorption of Ce6 and SiO_2-$P_{Ce6\text{-}IL2}$ at 410 nm was not decreased significantly after illumination for 1 and 2 min. The DPBF consumption was mainly caused by $^1O_2$.

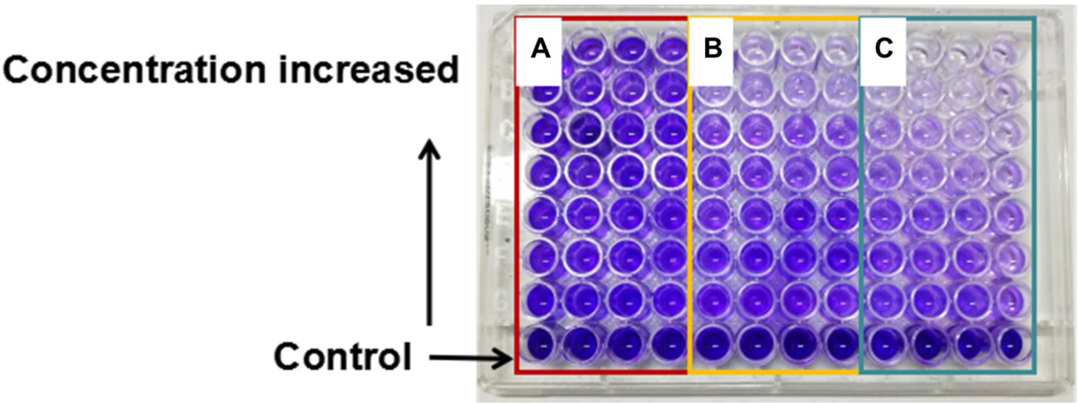

Figure S8. Semi quantitative analysis of MRSA biofilm elimination by crystal violet staining (A) Treated with Ce6. (B) Treated with SiO_2-$P_{Ce6-IL1}$. (C) Treated with SiO_2-$P_{Ce6-IL2}$.

20 μL different concentrations of Ce6, SiO_2-P_{Ce6-IL} interacted with biofilm for 10 s, and then illuminated for 15 min (5 mW/cm^2). After that, the residual biofilm was stained with 200 μL of 1.0% crystal violet solution for 30 min and 200 μL of ethanol was added to dissolve the crystal violet. The concentrations of Ce6, SiO_2-P_{Ce6-IL} were from 0 to 500 μM (0, 0.01, 0.05, 0.1 1.0, 50, 100 and 500 μM). After illumination for 15 min, the MRSA biofilm that treated with Ce6 was not eliminated at 100 μM, even at 500 μM. Compared with Ce6, the SiO_2-$P_{Ce6-IL2}$ could eliminate MRSA biofilm at 100 μM.

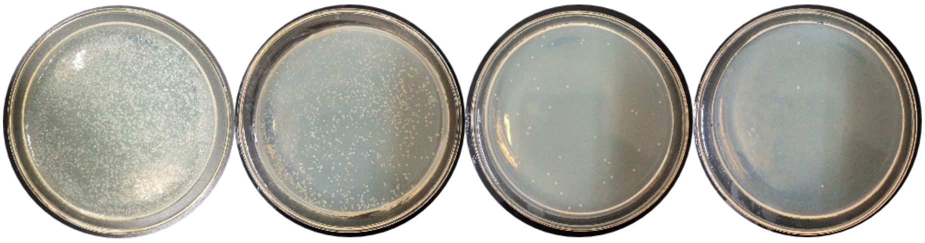

Figure S9. Photographs of agar plates for CFU counting of viable MRSA in biofilm grown in LB agar medium after treatment with SiO_2-$P_{Ce6\text{-}IL1}$ and SiO_2-$P_{Ce6\text{-}IL2}$, using Ce6 and PBS treatment as control.

The residual biofilms were dispersed under ultrasonication and the bacterial viability was analyzed by plate counting. The Figure S11 displayed the visual images of the agar plates and summarized the number of bacteria after treatment with Ce6, SiO_2-$P_{Ce6\text{-}IL}$ at 100 μM. The Ce6 alone cannot destroy the MRSA bacteria embedded in the biofilm.

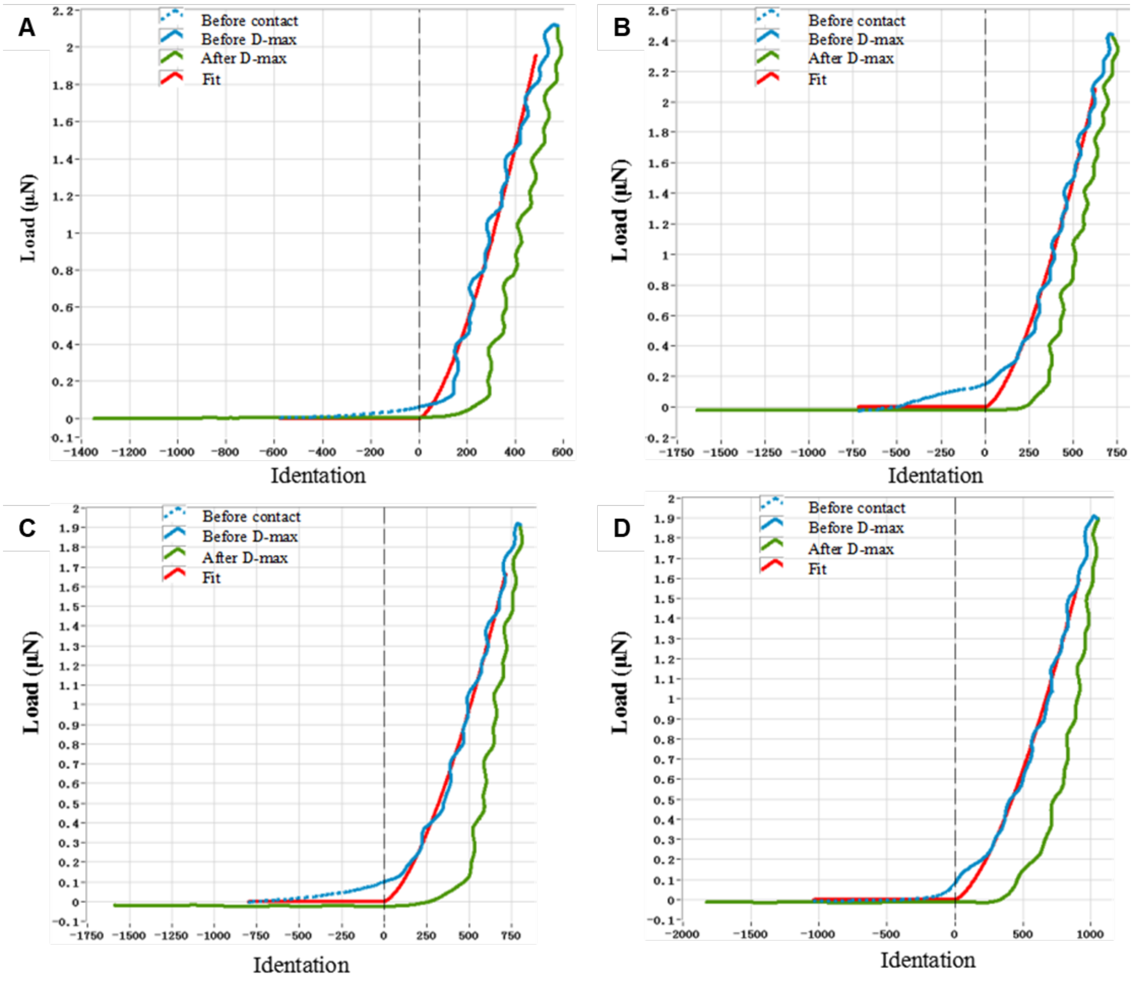

Figure S10. The mechanical properties of MRSA biofilm

(A) MRSA biofilm. (B) Treated with Ce6. (C) Treated with SiO_2-$P_{Ce6\text{-}IL1}$. (D) Treated with SiO_2-$P_{Ce6\text{-}IL2}$.

The nano indenter was used to examine the Young's modulus of biofilm. After treated with Ce6, SiO_2-$P_{Ce6\text{-}IL1}$ and SiO_2-$P_{Ce6\text{-}IL2,}$ the Young's modulus of biofilm was 435.29, 273.95, and 149.19 kpa, respectively. The mechanical properties treated with SiO_2-$P_{Ce6\text{-}IL2}$ was destroyed significantly.

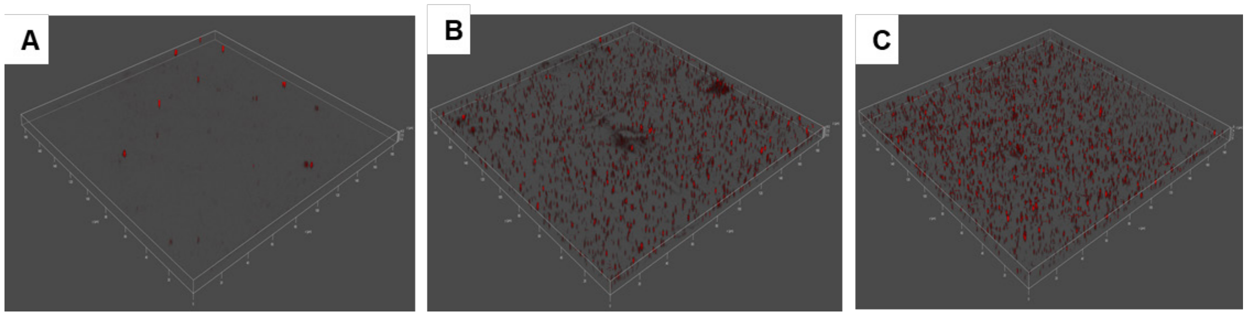

Figure S11. The ROS in the MRSA biofilm

(A) Treated with Ce6. (B) Treated with SiO_2-$P_{Ce6\text{-}IL1}$. (C) Treated with SiO_2-$P_{Ce6\text{-}IL2}$.

The ROS generation was detected by Cellular ROS Assay (deep red). As shown in Figure S14, only a very small amount of ROS was detected in Ce6 treatment. The high concentration of ROS treated by SiO_2-$P_{Ce6\text{-}IL}$ was observed in the biofilm.

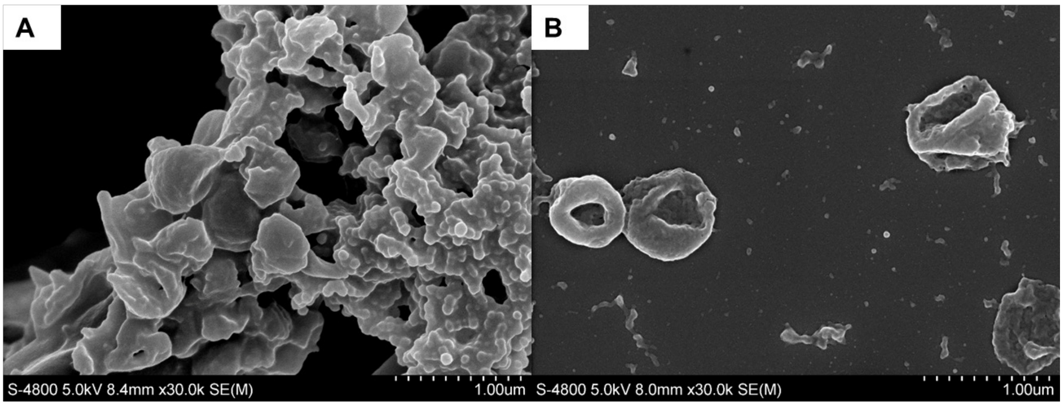

Figure S12. The morphology of the MRSA biofilm

(A) The MRSA biofilm treated by SiO_2-$P_{Ce6\text{-}IL1}$ with illumination for 15 min (5 mW/cm^2). (B) The MRSA biofilm treated by SiO_2-$P_{Ce6\text{-}IL2}$ with illumination for 15 min (5 mW/cm^2).

The structure and morphology MRSA biofilm was destroyed after treated by SiO_2-$P_{Ce6\text{-}IL1}$ or SiO_2-$P_{Ce6\text{-}IL2}$ with illumination for 15 min, especially for SiO_2-$P_{Ce6\text{-}IL2}$.
